# Postmarketing surveillance of elobixibat for patients with chronic constipation and concomitant schizophrenia or depression in Japan

**DOI:** 10.3389/fpsyt.2026.1763059

**Published:** 2026-04-20

**Authors:** Yoshiteru Takekita, Minami Umeyama, Mie Iwaida, Masaaki Higashikawa, Yusuke Shimada, Atsushi Nakajima

**Affiliations:** 1 Department of Neuropsychiatry, Faculty of Medicine, Kansai Medical University, Hirakata, Japan; 2 Medical Department, EA Pharma Co., Ltd., Tokyo, Japan; 3 Pharmaceutical Development Department, EA Pharma Co., Ltd., Tokyo, Japan; 4 Atami Hospital, International University of Health and Welfare, Atami, Japan

**Keywords:** constipation, depression, elobixibat, mental disorders, postmarketing surveillance, schizophrenia

## Abstract

**Background:**

Little is known about the optimal treatment for constipation in patients with schizophrenia or depression. Elobixibat is a laxative with a novel mechanism of action that inhibits the ileal bile acid transporter, acting as both an osmotic and a stimulant agent.

**Methods:**

We conducted a prospective, multicenter, postmarketing surveillance study to assess the safety and effectiveness of elobixibat for patients with chronic constipation in Japan (jRCT1080223950). The surveillance period was between June 2018 and May 2022. Patients were observed from the date of initial administration of elobixibat to 55 days thereafter (4-week treatment groups) or to 419 days thereafter (52-week treatment groups). Safety outcomes included adverse drug reactions (ADRs). Effectiveness outcomes included defecation frequency, Bristol Stool Form Scale (BSFS) scores, and constipation-related symptoms.

**Results:**

In the safety analysis set, the 4-week treatment groups comprised 105 patients with schizophrenia and 129 with depression; the 52-week treatment groups included 43 patients with schizophrenia and 55 with depression. Approximately 85% to 95% of patients used antipsychotics, and 40% to 55% used anxiolytics or sedative-hypnotics. The proportions of patients who experienced ADRs were 4.76% in the 4-week treatment group and 2.33% in the 52-week treatment group of patients with schizophrenia, and 3.88% and 9.09% of patients with depression. Diarrhea was the most common ADR in each group. There were no serious ADRs. In the 4-week treatment groups, the mean defecation frequency per week at baseline was 3.3 among patients with schizophrenia and 3.0 among patients with depression, which increased to 5.3 and 4.9, respectively, at week 4. In the 52-week treatment groups, the mean defecation frequency per week at week 52 was higher than that at baseline. After treatment, the proportion of patients with an ideal BSFS score of 4 increased in all groups by week 2 and reached approximately 60% by week 52. All constipation-related symptoms also improved by week 2 in all groups.

**Conclusions:**

Elobixibat improved chronic constipation with no new safety signal identified in patients with schizophrenia or depression and with available follow-up in real-world settings.

**Clinical trial registration:**

## 1 Introduction

Chronic constipation is one of the most prevalent gastrointestinal disorders, characterized by hard stools and symptoms, such as excessive straining and bloating, that last for months and impair the quality of life (QOL) of patients (1). An online survey targeting Japanese patients with chronic constipation demonstrated that both defecation frequency and stool form are relevant to QOL (2).

Schizophrenia is a severe mental disorder characterized by positive symptoms such as hallucinations, delusions, and disorganized behavior, as well as negative symptoms such as social withdrawal and diminished emotional expression (3). Depression is a mood disorder characterized by a feeling of sadness, loss of interest or pleasure, and a range of cognitive and physical symptoms that impair daily functioning in usual activities (4). Although the estimated lifetime prevalence of schizophrenia is reported to be lower than that of depression in Japan (5, 6), more patients with schizophrenia require hospitalization (7).

An association between constipation and depression has been recognized (8–10). In a database study in Japan, the prevalence of constipation doubled in individuals with depression or anxiety, or both (9). The prevalence of constipation was reported to be increased in patients with schizophrenia (11, 12). Some psychiatric medications have anticholinergic effects (13), which may reduce intestinal motility and induce constipation in some patients (14). A questionnaire survey targeting patients with schizophrenia found that the doses of antiparkinsonian drugs (all, except for amantadine, had anticholinergic effects in the study) and benzodiazepine hypnotics were associated with constipation (11). In a cross-sectional study of patients with schizophrenia, the use of anticholinergics, aripiprazole, and quetiapine was identified as a risk factor for constipation (12). A recent genome-wide association study in a European population showed genetic overlap between schizophrenia and constipation (15).

Elobixibat is a laxative with a novel mechanism of action that inhibits the ileal bile acid transporter and increases the delivery of bile acids to the colon (16). Because bile acids increase colonic secretion of water and electrolytes (17) and stimulate high-amplitude, propagated contractions (18), elobixibat acts as both an osmotic and a stimulant agent. A recent single-center study also showed that elobixibat improved defecation desire in patients with chronic constipation (19). A phase 3 trial of elobixibat among patients in Japan with chronic constipation demonstrated its onset of action within 2 weeks and long-term safety up to 52 weeks (20). Recent multicenter, postmarketing surveillance of patients with chronic constipation in Japan confirmed the real-world safety and effectiveness of elobixibat in patients with chronic constipation, including those aged ≥65 years (21). However, little is known about the safety and effectiveness of elobixibat for treating chronic constipation among patients with schizophrenia or depression.

In the present report, we conducted a subgroup analysis of the postmarketing surveillance using data from patients who had schizophrenia or depression as comorbidities. We assessed the safety and effectiveness of elobixibat in this cohort, as well as the status of concomitant psychiatric medication use.

## 2 Materials and methods

### 2.1 Study design and patients

The prospective, multicenter, postmarketing surveillance study assessed the safety and effectiveness of elobixibat for patients with chronic constipation in Japan (21). The surveillance period was between June 2018 and May 2022. The observation period was set from the date of initial administration of elobixibat to 55 days thereafter for the 4-week treatment group, and to 419 days thereafter for the 52-week treatment group. The 52-week treatment group included patients who continued treatment beyond the initial 4-week period.

Patients were enrolled if they were diagnosed with chronic constipation and were prescribed elobixibat for the first time. In the present report, we conducted a subgroup analysis of postmarketing surveillance using data from patients whose electronic case report forms listed schizophrenia or depression as a comorbidity. The diagnosis of schizophrenia or depression was made by certified psychiatrists, but not based on the study-specific criteria. Elobixibat was given orally as a 10-mg dose once daily before a meal, which could be adjusted to a 5- or 15-mg dose based on patient symptoms. Baseline patient characteristics, treatment status, any medications concomitantly used with elobixibat during the observation period, any adverse events (AEs), and constipation-related symptoms were collected from the electronic case report forms.

The surveillance was conducted in accordance with the Declaration of Helsinki and its latest revision (2013) and Good Postmarketing Study Practice (GPSP) as outlined in the Ministry of Health, Labour and Welfare Ordinance No. 171, dated December 20, 2004. Although GPSP does not regulate ethical approval and informed consent, the study protocol was reviewed and approved by the Pharmaceuticals and Medical Devices Agency before the study was initiated, and informed consent had been obtained verbally from all participants before their enrollment. This study was registered with the Japan Registry of Clinical Trials (jRCT1080223950).

### 2.2 Safety assessments

All AEs were coded to preferred terms (PTs) of the ICH Medical Dictionary for Regulatory Activities/Japanese (MedDRA/J) version 25.1. Adverse drug reactions (ADRs) were AEs whose causal relationship with elobixibat could not be ruled out based on the assessment of investigators at each participating institution. If the same event occurred multiple times in the same patient during the observation period (i.e., from the date of initial administration of elobixibat to 55 days thereafter for the 4-week treatment group and to 419 days thereafter for the 52-week treatment group), only the first event was counted.

### 2.3 Effectiveness assessments

Investigators interviewed patients regarding their defecation using questionnaires (21) at baseline and weeks 2, 4, 12, 24, 36, and 52 after initial treatment. Efficacy outcomes included weekly defecation frequency (i.e., the number of defecations during the 7 days before each assessment), Bristol Stool Form Scale (BSFS) scores, bloating, straining during defecation, defecation satisfaction, and time to defecation after elobixibat administration (most recent event).

### 2.4 Statistical analyses

The safety analysis set included all patients in this subgroup analysis who received elobixibat and had safety information, excluding those previously treated with elobixibat at enrollment. The effectiveness analysis set included patients in the safety analysis set, excluding cases without efficacy data, those at institutions excluded from the study, and those with off-label use.

Descriptive statistics are used to summarize continuous data as mean (standard deviation [SD], 95% confidence interval [CI], or both) and categorical data as n (%). Missing data were not imputed, and only available data were used for the analysis. For exploratory purposes, defecation frequency and BSFS scores were compared pairwise between baseline and each subsequent assessment using a paired *t*-test or a Wilcoxon signed-rank test, respectively. Multiple comparison correction was not performed to adjust *p*-values. The significance level was set at a *p*-value of 5% (two-tailed). As a sensitivity analysis, a mixed model for repeated measures (MMRM) analysis was performed for defecation frequency under the assumption that missing data were missing at random. All statistical analyses were performed using SAS release 9.4 (SAS Institute Japan, Tokyo, Japan).

## 3 Results

### 3.1 Baseline patient characteristics

In the previous report, safety was analyzed in the 4-week treatment group of 3, 638 patients and the 52-week treatment group of 1, 315 patients (21). In this report, safety was analyzed in the 4-week treatment group of 105 patients with schizophrenia and 129 patients with depression, as well as in the 52-week treatment group of 43 patients with schizophrenia and 55 patients with depression (**Supplementary Figure 1**).

In the 4-week treatment groups, women accounted for 56.2% (59/105) of patients with schizophrenia and 69.0% (89/129) of those with depression (**Table 1**). No women were pregnant. Individuals aged ≥65 years accounted for 42.9% (45/105) of patients with schizophrenia and 63.6% (82/129) of those with depression. The proportion of inpatients among patients with schizophrenia (49.5% [52/105]) was higher than that for patients with depression (10.9% [14/129]). Approximately half of the patients had chronic constipation for ≥5 years. The proportions of patients with prior prescription laxative use and concomitant laxative use were 70.5% (74/105) and 39.0% (41/105) for patients with schizophrenia, and 79.8% (103/129) and 45.7% (59/129) for patients with depression. The most common prior prescription laxatives, as well as the most common concomitant laxatives, included saline laxatives and stimulant laxatives in both patient groups. Prior and concomitant use of stimulant laxatives was more prevalent among patients with schizophrenia than those with depression.

**Table 1:** Baseline patient characteristics in the safety analysis set.

	Patients with schizophrenia		Patients with depression	
Items	4-week treatment N = 105	52-week treatment N = 43	4-week treatment N = 129	52-week treatment N = 55
Sex				
Men	46 (43.8)	19 (44.2)	40 (31.0)	17 (30.9)
Women	59 (56.2)	24 (55.8)	89 (69.0)	38 (69.1)
Pregnant^a^				
Yes	0 (0.0)	0 (0.0)	0 (0.0)	0 (0.0)
Age, years				
< 65	60 (57.1)	24 (55.8)	47 (36.4)	16 (29.1)
≥ 65	45 (42.9)	19 (44.2)	82 (63.6)	39 (70.9)
BMI, kg/m^2^				
< 18.5	9 (8.6)	4 (9.3)	12 (9.3)	7 (12.7)
18.5 to < 25	43 (41.0)	14 (32.6)	56 (43.4)	26 (47.3)
≥ 25	14 (13.3)	4 (9.3)	13 (10.1)	5 (9.1)
Unknown	39 (37.1)	21 (48.8)	48 (37.2)	17 (30.9)
Outpatient/inpatient				
Outpatient	53 (50.5)	17 (39.5)	115 (89.1)	48 (87.3)
Inpatient	52 (49.5)	26 (60.5)	14 (10.9)	7 (12.7)
Duration of chronic constipation, years				
< 5	36 (34.3)	15 (34.9)	52 (40.3)	19 (34.5)
≥ 5	59 (56.2)	25 (58.1)	56 (43.4)	31 (56.4)
Unknown	10 (9.5)	3 (7.0)	21 (16.3)	5 (9.1)
IBS-C				
Yes	9 (8.6)	4 (9.3)	16 (12.4)	6 (10.9)
Prior OTC laxative use				
Yes	2 (1.9)	1 (2.3)	9 (7.0)	3 (5.5)
Prior prescription laxative use, yes^b^				
Any	74 (70.5)	33 (76.7)	103 (79.8)	44 (80.0)
Saline laxatives^c^	36 (48.6)	15 (45.5)	58 (56.3)	21 (47.7)
Sugar-like laxatives^c^	1 (1.4)	0 (0.0)	2 (1.9)	0 (0.0)
PEG preparations^c^	4 (5.4)	1 (3.0)	1 (1.0)	1 (2.3)
Intestinal secretagogues^c^	14 (18.9)	5 (15.2)	19 (18.4)	10 (22.7)
Bulk-forming laxatives^c^	1 (1.4)	1 (3.0)	4 (3.9)	2 (4.5)
Stimulant laxatives^c^	49 (66.2)	27 (81.8)	44 (42.7)	19 (43.2)
Other^c^	14 (18.9)	7 (21.2)	37 (35.9)	16 (36.4)
Concomitant laxative use, yes^b^				
Any	41 (39.0)	18 (41.9)	59 (45.7)	31 (56.4)
Saline laxatives^d^	23 (56.1)	11 (61.1)	30 (50.8)	17 (54.8)
Sugar-like laxatives^d^	0 (0.0)	0 (0.0)	1 (1.7)	0 (0.0)
PEG preparations^d^	1 (2.4)	1 (5.6)	2 (3.4)	2 (6.5)
Intestinal secretagogues^d^	7 (17.1)	5 (27.8)	8 (13.6)	6 (19.4)
Bulk-forming laxatives^d^	1 (2.4)	1 (5.6)	2 (3.4)	1 (3.2)
Stimulant laxatives^d^	25 (61.0)	13 (72.2)	20 (33.9)	10 (32.3)
Other^d,e^	11 (26.8)	6 (33.3)	28 (47.5)	12 (38.7)

BMI, body mass index; IBS-C, irritable bowel syndrome with constipation; OTC, over-the-counter; PEG, polyethylene glycol.

Values are shown as n (%).

a For women.

b Multiple responses are allowed.

c The proportion shown refers to that among patients who used any prior prescribed laxatives.

d The proportion shown refers to that among patients who used any concomitant laxatives.

e Including Kampo medicines and probiotics.

Baseline characteristics of the 52-week treatment groups were similar to those of the 4-week treatment groups, except for the following items. Among patients with schizophrenia, the proportion of inpatients was higher in the 52-week treatment group than in the 4-week treatment group. Among patients with depression, the proportion of patients with a disease duration of ≥5 years was higher in the 52-week treatment group than in the 4-week treatment group.

### 3.2 Treatment status

The most common maximum daily dose of elobixibat was 10 mg in all groups (**Table 2**). The proportion of patients treated with 15 mg elobixibat was higher in the 52-week treatment group than in the 4-week treatment group among patients with schizophrenia (18.6% [8/43] vs. 12.4% [13/105]) as well as those with depression (20.0% [11/55] vs. 9.3% [12/129]). In the 4-week treatment groups, the treatment continuation rate was approximately 80%, with lack of efficacy being the most common reason for treatment discontinuation or termination. In the 52-week treatment groups, patients with depression had a lower treatment continuation rate (50.9% [28/55] vs. 72.1% [31/43]) and a higher drop-out rate (14.5% [8/55] vs. 4.7% [2/43]) than those with schizophrenia. **Supplementary Table 1** shows a descriptive comparison between those who completed the 52-week treatment and those who discontinued treatment. Among patients with schizophrenia, those who discontinued treatment were more likely to be < 65 years, have irritable bowel syndrome with constipation (IBS-C), and have prior over-the-counter (OTC) laxative use than those who completed treatment. Among patients with depression, those who discontinued treatment were more likely to have IBS-C, prior OTC laxative use, and concomitant laxative use than those who completed treatment.

**Table 2:** Treatment status in the safety analysis set.

	Patients with schizophrenia		Patients with depression	
Items	4-week treatment N = 105	52-week treatment N = 43	4-week treatment N = 129	52-week treatment N = 55
Maximum daily dose				
1 tablet (5 mg)	15 (14.3)	4 (9.3)	10 (7.8)	3 (5.5)
2 tablets (10 mg)	77 (73.3)	31 (72.1)	107 (82.9)	41 (74.5)
3 tablets (15 mg)	13 (12.4)	8 (18.6)	12 (9.3)	11 (20.0)
Treatment persistence				
Continuing	82 (78.1)	31 (72.1)	105 (81.4)	28 (50.9)
Discontinued or terminated	23 (21.9)	12 (27.9)	24 (18.6)	27 (49.1)
Reasons for treatment discontinuation or termination				
Symptom improvement	6 (5.7)	3 (7.0)	3 (2.3)	6 (10.9)
Lack of efficacy	8 (7.6)	3 (7.0)	9 (7.0)	2 (3.6)
AEs	5 (4.8)	1 (2.3)	5 (3.9)	5 (9.1)
Patient request (except AEs)	2 (1.9)	3 (7.0)	4 (3.1)	6 (10.9)
Dropping out during treatment	2 (1.9)	2 (4.7)	2 (1.6)	8 (14.5)
Other	0 (0.0)	0 (0.0)	1 (0.8)	0 (0.0)

AEs, adverse events.

Values are shown as n (%).

### 3.3 Safety

The proportions of patients who experienced ADRs were 4.76% (5/105) in the 4-week treatment group and 2.33% (1/43) in the 52-week treatment group of patients with schizophrenia, and 3.88% (5/129) and 9.09% (5/55) of patients with depression (**Table 3**). None of the ADRs were serious. The most common ADRs were gastrointestinal disorders, including diarrhea.

**Table 3:** Adverse drug reactions in the safety analysis set.

	Patients with schizophrenia		Patients with depression	
Items	4-week treatment N = 105	52-week treatment N = 43	4-week treatment N = 129	52-week treatment N = 55
Patients with any adverse drug reaction	5 (4.76)	1 (2.33)	5 (3.88)	5 (9.09)
Details of adverse drug reactions				
Cardiac disorders	0 (0.0)	0 (0.0)	0 (0.0)	1 (1.82)^b^
Cardiac failure	0 (0.0)	0 (0.0)	0 (0.0)	1 (1.82)^b^
Vascular disorders	0 (0.0)	0 (0.0)	0 (0.0)	1 (1.82)^b^
Hypertension	0 (0.0)	0 (0.0)	0 (0.0)	1 (1.82)^b^
Gastrointestinal disorders	5 (4.76)	1 (2.33)^a^	5 (3.88)	3 (5.45)^a^
Abdominal discomfort	1 (0.95)	0 (0.0)	0 (0.0)	0 (0.0)
Abdominal distension	1 (0.95)	0 (0.0)	0 (0.0)	0 (0.0)
Abdominal pain	0 (0.0)	0 (0.0)	1 (0.78)	0 (0.0)
Diarrhea	3 (2.86)	1 (2.33)^a^	3 (2.33)	3 (5.45)^a^
Soft feces	0 (0.0)	0 (0.0)	1 (0.78)	0 (0.0)
Hepatobiliary disorders	0 (0.0)	0 (0.0)	0 (0.0)	1 (1.82)^b^
Hepatic function abnormal	0 (0.0)	0 (0.0)	0 (0.0)	1 (1.82)^b^

Values are shown as n (%).

Adverse drug reactions were coded using a preferred term and classified by System Organ Class according to the International Conference on Harmonisation of Technical Requirements for Registration of Pharmaceuticals for Human Use (ICH) Medical Dictionary for Regulatory Activities, Japanese version 25.1.

a Adverse drug reactions that occurred during the 4-week treatment period.

b Adverse drug reactions that occurred in a single patient after the 4-week treatment period.

### 3.4 Effectiveness

#### 3.4.1 Defecation frequency

**Figure 1:**
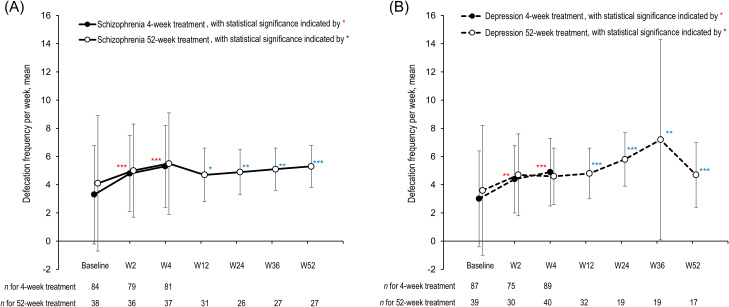
Change in defecation frequency per week. Plots indicate the mean defecation frequency per week for patients with schizophrenia **(A)** (solid lines) and those with depression **(B)** (dashed lines). Data for the 4-week treatment groups and 52-week groups are shown as black circles and white circles, respectively. Error bars indicate standard deviations. n, number of patients; W, week. ^*^p < 0.05, ^**^p < 0.01, ^***^p < 0.001 versus baseline (red asterisks for the 4-week treatment groups and blue asterisks for the 52-week treatment groups).

In the previous report, effectiveness was analyzed in the 4-week treatment group of 3, 410 patients and the 52-week treatment group of 1, 215 patients (21). In the present report, effectiveness was analyzed in the 4-week treatment group of 100 patients with schizophrenia and 120 patients with depression, as well as in a 52-week treatment group of 42 patients with schizophrenia and 51 patients with depression (**Supplementary Figure 1**). Among patients with schizophrenia (**Figure 1A**), the mean defecation frequency per week in the 4-week treatment group was 3.3 (SD 3.5, 95% CI 2.53–4.04) at baseline, which increased to 4.8 (SD 2.7, 95% CI 4.25–5.45) at week 2 (nominal p < 0.001) and 5.3 (SD 2.9, 95% CI 4.67–5.97) at week 4 (nominal p < 0.001). Among patients with depression (**Figure 1B**), the mean defecation frequency per week in the 4-week treatment group was 3.0 (SD 3.4, 95% CI 2.30–3.75) at baseline, which increased to 4.4 (SD 2.4, 95% CI 3.86–4.97) at week 2 (nominal p < 0.01) and 4.9 (SD 2.4, 95% CI 4.37–5.40) at week 4 (nominal p < 0.001). In the 52-week treatment groups, the mean defecation frequency improved from baseline at week 12 and thereafter (**Figures 1A, B**). Detailed information on the defecation frequency per week are summarized in **Supplementary Table 2** (mean, SD, and 95% CI calculated from observed data; least squares mean, standard error, and 95% CI in an MMRM analysis), and **Supplementary Figure 2** (comparison between observed data and MMRM estimates). The findings obtained from the MMRM analysis align with those from the primary analysis.

#### 3.4.2 Bristol Stool Form Scale scores

**Figure 2:**
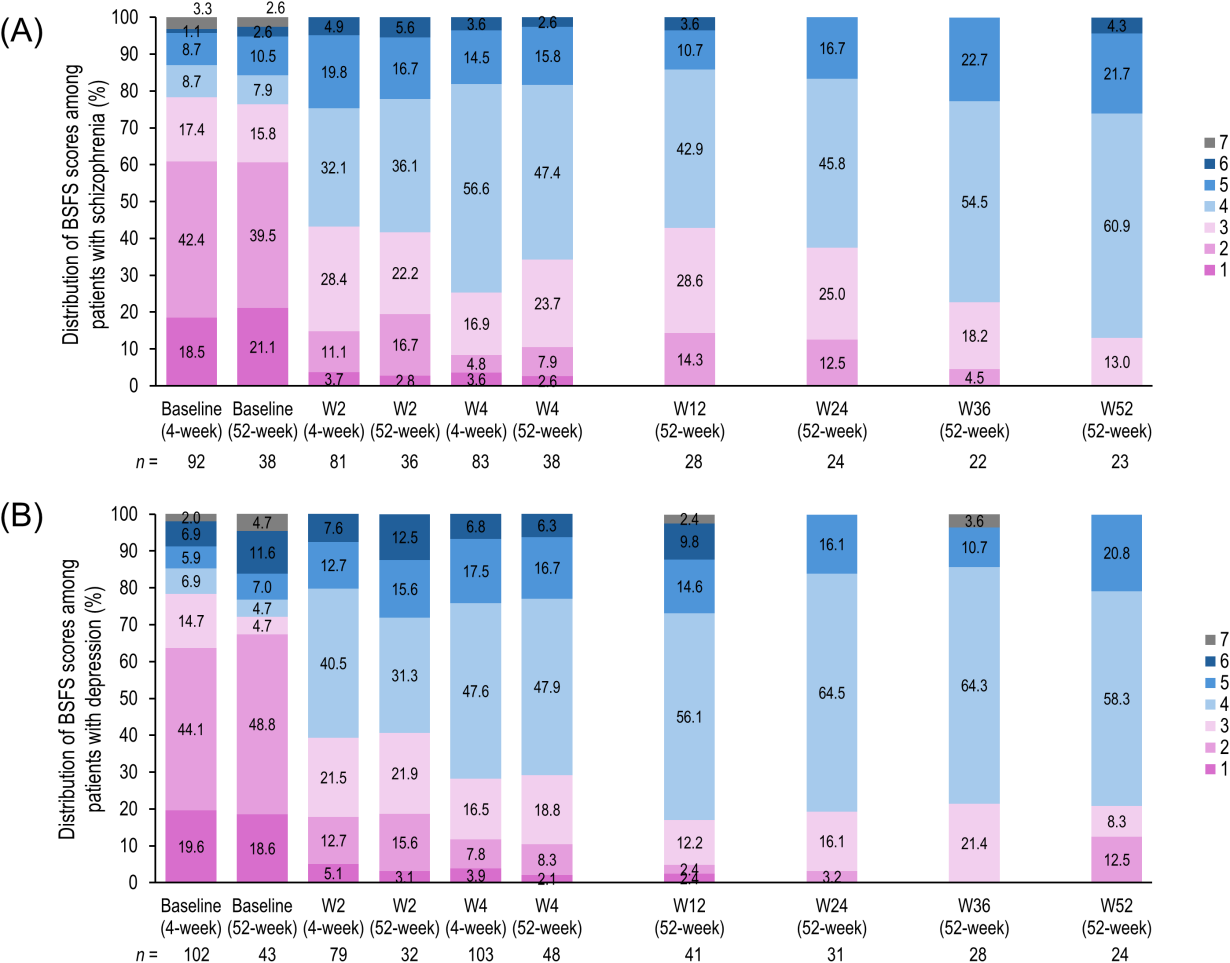
Change in the distribution of Bristol Stool Form Scale scores. Stacked bar graphs indicating the distribution of BSFS scores for patients with schizophrenia **(A)** and those with depression **(B)**. BSFS scores range from the hardest (type 1) to the loosest (type 7) stool, with type 4 being the ideal stool form. Color coded key indicates BSFS score. BSFS, Bristol Stool Form Scale; n, number of patients; W, week.

At baseline, 70% to 80% of patients in this subgroup analysis had a BSFS score of ≤3 (**Figures 2A, B**). After treatment, the proportion of patients with an ideal BSFS score of 4 increased as early as week 2 and reached approximately 60% by week 52. The mean BSFS scores also increased as early as week 2 and remained significantly higher than baseline at subsequent assessments (**Table 4**).

**Table 4:** Change in BSFS scores in the effectiveness analysis set.

Items	Baseline	Week 2	Week 4	Week 12	Week 24	Week 36	Week 52
Patients with schizophrenia							
4-week treatment group	n = 92	n = 81	n = 83	–	–	–	–
Mean (SD)	2.6 (1.5)	3.7 (1.2)^***^	3.8 (1.0)^***^	–	–	–	–
52-week treatment group	n = 38	n = 36	n = 38	n = 28	n = 24	n = 22	n = 23
Mean (SD)	2.7 (1.5)	3.6 (1.2)^***^	3.7 (1.0)^***^	3.6 (1.0)^**^	3.7 (0.9)^*^	4.0 (0.8)^**^	4.2 (0.7)^***^
Patients with depression							
4-week treatment group	n = 102	n = 79	n = 103	–	–	–	–
Mean (SD)	2.6 (1.5)	3.7 (1.2)^***^	3.9 (1.1)^***^	–	–	–	–
52-week treatment group	n = 43	n = 32	n = 48	n = 41	n = 31	n = 28	n = 24
Mean (SD)	2.9 (1.8)	3.8 (1.3)^**^	3.9 (1.1)^***^	4.2 (1.1)^***^	3.9 (0.7)^***^	4.0 (0.8)^***^	3.9 (0.9)^**^

^*^p < 0.05, ^**^p < 0.01, ^***^p < 0.001 compared with baseline.

BSFS, Bristol Stool Form Scale; SD, standard deviation.

#### 3.4.3 Constipation-related symptoms

In the 4-week treatment groups, the proportion of patients with schizophrenia always or frequently experiencing bloating or straining during defecation at baseline was 38.5% (35/91) and 51.1% (45/88), respectively, which decreased to 12.2% (10/82) and 9.9% (8/81) at week 2 and remained lower than baseline at subsequent assessments (**Table 5**). The proportion of patients who were satisfied or slightly satisfied with their defecation at baseline was 6.5% (6/92), which increased to 67.5% (54/80) at week 2 and remained higher than baseline at subsequent assessments. Among patients with depression, the proportion of patients always or frequently experiencing bloating or straining during defecation at baseline was 38.7% (41/106) and 53.8% (56/104), respectively, which decreased to 17.3% (14/81) and 19.0% (15/79) at week 2 and remained lower than baseline at subsequent assessments (**Table 6**). The proportion of patients who were satisfied or slightly satisfied with their defecation at baseline was 5.4% (6/112), which increased to 60.5% (49/81) at week 2 and remained higher than baseline at subsequent assessments. The changes in constipation-related symptoms in the 52-week treatment groups were similar to those in the 4-week treatment groups (**Tables 5**, **6**). The mean time to defecation after elobixibat administration was < 8 hours, with > 70% of patients having defecation within 24 h (**Tables 5**, **6**).

**Table 5:** Change in constipation-related symptoms among patients with schizophrenia in the effectiveness analysis set.

Items	Baseline	Week 2	Week 4	Week 12	Week 24	Week 36	Week 52
4-week treatment							
Bloating	n = 91	n = 82	n = 85	–	–	–	–
Never	5 (5.5)	16 (19.5)	23 (27.1)	–	–	–	–
Rarely	12 (13.2)	27 (32.9)	34 (40.0)	–	–	–	–
Occasionally	39 (42.9)	29 (35.4)	24 (28.2)	–	–	–	–
Frequently	28 (30.8)	6 (7.3)	2 (2.4)	–	–	–	–
Always	7 (7.7)	4 (4.9)	2 (2.4)	–	–	–	–
Straining during defecation	n = 88	n = 81	n = 83	–	–	–	–
Never	5 (5.7)	16 (19.8)	19 (22.9)	–	–	–	–
Rarely	9 (10.2)	27 (33.3)	36 (43.4)	–	–	–	–
Occasionally	29 (33.0)	30 (37.0)	25 (30.1)	–	–	–	–
Frequently	38 (43.2)	7 (8.6)	1 (1.2)	–	–	–	–
Always	7 (8.0)	1 (1.2)	2 (2.4)	–	–	–	–
Defecation satisfaction	n = 92	n = 80	n = 84	–	–	–	–
Satisfied	2 (2.2)	20 (25.0)	32 (38.1)	–	–	–	–
Slightly satisfied	4 (4.3)	34 (42.5)	38 (45.2)	–	–	–	–
Slightly unsatisfied	33 (35.9)	15 (18.8)	10 (11.9)	–	–	–	–
Unsatisfied	53 (57.6)	11 (13.8)	4 (4.8)	–	–	–	–
Time to defecation after elobixibat administration	–	n = 40	n = 38	–	–	–	–
Mean hours (SD), n	–	7.5 (5.9), 30	7.8 (5.5), 28	–	–	–	–
≤ 24 hours	–	30 (75.0)	28 (73.7)	–	–	–	–
≤ 48 hours	–	30 (75.0)	28 (73.7)	–	–	–	–
52-week treatment							
Bloating	n = 38	n = 36	n = 38	n = 30	n = 27	n = 25	n = 26
Never	3 (7.9)	6 (16.7)	8 (21.1)	7 (23.3)	10 (37.0)	9 (36.0)	9 (34.6)
Rarely	1 (2.6)	12 (33.3)	14 (36.8)	13 (43.3)	10 (37.0)	10 (40.0)	12 (46.2)
Occasionally	18 (47.4)	13 (36.1)	15 (39.5)	8 (26.7)	6 (22.2)	5 (20.0)	4 (15.4)
Frequently	14 (36.8)	3 (8.3)	0 (0.0)	1 (3.3)	0 (0.0)	0 (0.0)	0 (0.0)
Always	2 (5.3)	2 (5.6)	1 (2.6)	1 (3.3)	1 (3.7)	1 (4.0)	1 (3.8)
Straining during defecation	n = 38	n = 36	n = 38	n = 30	n = 27	n = 25	n = 26
Never	4 (10.5)	5 (13.9)	7 (18.4)	5 (16.7)	7 (25.9)	6 (24.0)	7 (26.9)
Rarely	4 (10.5)	15 (41.7)	17 (44.7)	14 (46.7)	11 (40.7)	13 (52.0)	14 (53.8)
Occasionally	13 (34.2)	14 (38.9)	12 (31.6)	9 (30.0)	8 (29.6)	5 (20.0)	4 (15.4)
Frequently	15 (39.5)	2 (5.6)	1 (2.6)	2 (6.7)	0 (0.0)	0 (0.0)	0 (0.0)
Always	2 (5.3)	0 (0.0)	1 (2.6)	0 (0.0)	1 (3.7)	1 (4.0)	1 (3.8)
Defecation satisfaction	n = 39	n = 35	n = 39	n = 33	n = 29	n = 27	n = 28
Satisfied	1 (2.6)	12 (34.3)	13 (33.3)	9 (27.3)	8 (27.6)	7 (25.9)	10 (35.7)
Slightly satisfied	2 (5.1)	12 (34.3)	21 (53.8)	17 (51.5)	17 (58.6)	17 (63.0)	16 (57.1)
Slightly unsatisfied	16 (41.0)	8 (22.9)	3 (7.7)	4 (12.1)	3 (10.3)	2 (7.4)	2 (7.1)
Unsatisfied	20 (51.3)	3 (8.6)	2 (5.1)	3 (9.1)	1 (3.4)	1 (3.7)	0 (0.0)
Time to defecation after elobixibat administration	–	n = 18	n = 20	n = 18	n = 13	n = 14	n = 13
Mean hours (SD), n	–	5.0 (2.2), 15	5.9 (2.4), 16	6.6 (3.6), 18	6.0 (3.5), 12	6.2 (3.4), 14	5.7 (3.4), 13
≤ 24 hours	–	15 (83.3)	16 (80.0)	18 (100.0)	12 (92.3)	14 (100.0)	13 (100.0)
≤ 48 hours	–	15 (83.3)	16 (80.0)	18 (100.0)	12 (92.3)	14 (100.0)	13 (100.0)

Values are shown as n (%).

**Table 6:** Change in constipation-related symptoms among patients with depression in the effectiveness analysis set.

Items	Baseline	Week 2	Week 4	Week 12	Week 24	Week 36	Week 52
4-week treatment							
Bloating	n = 106	n = 81	n = 101	–	–	–	–
Never	9 (8.5)	18 (22.2)	32 (31.7)	–	–	–	–
Rarely	21 (19.8)	26 (32.1)	36 (35.6)	–	–	–	–
Occasionally	35 (33.0)	23 (28.4)	20 (19.8)	–	–	–	–
Frequently	28 (26.4)	10 (12.3)	7 (6.9)	–	–	–	–
Always	13 (12.3)	4 (4.9)	6 (5.9)	–	–	–	–
Straining during defecation	n = 104	n = 79	n = 97	–	–	–	–
Never	7 (6.7)	14 (17.7)	14 (14.4)	–	–	–	–
Rarely	7 (6.7)	23 (29.1)	39 (40.2)	–	–	–	–
Occasionally	34 (32.7)	27 (34.2)	30 (30.9)	–	–	–	–
Frequently	34 (32.7)	10 (12.7)	6 (6.2)	–	–	–	–
Always	22 (21.2)	5 (6.3)	8 (8.2)	–	–	–	–
Defecation satisfaction	n = 112	n = 81	n = 105	–	–	–	–
Satisfied	0 (0.0)	15 (18.5)	29 (27.6)	–	–	–	–
Slightly satisfied	6 (5.4)	34 (42.0)	39 (37.1)	–	–	–	–
Slightly unsatisfied	30 (26.8)	18 (22.2)	18 (17.1)	–	–	–	–
Unsatisfied	76 (67.9)	14 (17.3)	19 (18.1)	–	–	–	–
Time to defecation after elobixibat administration	–	n = 50	n = 63	–	–	–	–
Mean hours (SD), n	–	6.4 (4.9), 35	7.1 (5.8), 47	–	–	–	–
≤ 24 hours	–	35 (70.0)	47 (74.6)	–	–	–	–
≤ 48 hours	–	35 (70.0)	47 (74.6)	–	–	–	–
52-week treatment							
Bloating	n = 46	n = 33	n = 47	n = 42	n = 31	n = 29	n = 25
Never	4 (8.7)	10 (30.3)	14 (29.8)	12 (28.6)	11 (35.5)	8 (27.6)	7 (28.0)
Rarely	11 (23.9)	9 (27.3)	16 (34.0)	16 (38.1)	13 (41.9)	14 (48.3)	10 (40.0)
Occasionally	15 (32.6)	10 (30.3)	12 (25.5)	11 (26.2)	5 (16.1)	4 (13.8)	4 (16.0)
Frequently	10 (21.7)	3 (9.1)	3 (6.4)	1 (2.4)	1 (3.2)	1 (3.4)	3 (12.0)
Always	6 (13.0)	1 (3.0)	2 (4.3)	2 (4.8)	1 (3.2)	2 (6.9)	1 (4.0)
Straining during defecation	n = 45	n = 31	n = 43	n = 42	n = 32	n = 29	n = 26
Never	4 (8.9)	5 (16.1)	4 (9.3)	12 (28.6)	10 (31.3)	9 (31.0)	7 (26.9)
Rarely	4 (8.9)	8 (25.8)	18 (41.9)	15 (35.7)	14 (43.8)	12 (41.4)	12 (46.2)
Occasionally	14 (31.1)	11 (35.5)	13 (30.2)	13 (31.0)	6 (18.8)	6 (20.7)	4 (15.4)
Frequently	15 (33.3)	4 (12.9)	3 (7.0)	1 (2.4)	1 (3.1)	1 (3.4)	2 (7.7)
Always	8 (17.8)	3 (9.7)	5 (11.6)	1 (2.4)	1 (3.1)	1 (3.4)	1 (3.8)
Defecation satisfaction	n = 50	n = 33	n = 49	n = 41	n = 30	n = 28	n = 25
Satisfied	0 (0.0)	5 (15.2)	11 (22.4)	20 (48.8)	19 (63.3)	16 (57.1)	15 (60.0)
Slightly satisfied	3 (6.0)	15 (45.5)	21 (42.9)	14 (34.1)	8 (26.7)	7 (25.0)	5 (20.0)
Slightly unsatisfied	13 (26.0)	9 (27.3)	8 (16.3)	5 (12.2)	2 (6.7)	3 (10.7)	3 (12.0)
Unsatisfied	34 (68.0)	4 (12.1)	9 (18.4)	2 (4.9)	1 (3.3)	2 (7.1)	2 (8.0)
Time to defecation after elobixibat administration	–	n = 20	n = 30	n = 18	n = 10	n = 9	n = 7
Mean hours (SD), n	–	4.5 (2.7), 15	5.3 (5.1), 23	4.5 (2.7), 17	3.6 (2.4), 10	3.9 (2.5), 9	4.3 (2.3), 7
≤ 24 hours	–	15 (75.0)	23 (76.7)	17 (94.4)	10 (100.0)	9 (100.0)	7 (100.0)
≤ 48 hours	–	15 (75.0)	23 (76.7)	17 (94.4)	10 (100.0)	9 (100.0)	7 (100.0)

Values are shown as n (%).

#### 3.4.4 Concomitant psychiatric medications

In the safety analysis set, approximately 85% to 95% of patients used antipsychotics or antidepressants, and 40% to 55% used anxiolytics or sedative-hypnotics (**Table 7**). Almost all of the anxiolytics and 60%-70% of the hypnotics were benzodiazepines. Among patients with schizophrenia, the most commonly used psychiatric medications included second generation antipsychotics (with or without anticholinergic effects), hypnotics, and first generation antipsychotics with anticholinergic effects. Among patients with depression, the most commonly used psychiatric medications included hypnotics, noradrenergic and specific serotonergic antidepressants (NaSSAs), and selective serotonin reuptake inhibitors (SSRIs) for the 4-week treatment group and hypnotics, NaSSAs, SSRIs, and second generation antipsychotics with anticholinergic effects for the 52-week treatment group.

**Table 7:** Concomitant psychiatric medications in the safety analysis set.

Items	Patients with schizophrenia		Patients with depression	
	4-week treatment N = 105	52-week treatment N = 43	4-week treatment N = 129	52-week treatment N = 55
Antipsychotics or antidepressants	92 (87.6)	41 (95.3)	110 (85.3)	51 (92.7)
Antipsychotics				
First-generation antipsychotics with anticholinergic effects^a^	22 (21.0)	11 (25.6)	4 (3.1)	2 (3.6)
First-generation antipsychotics without anticholinergic effects	5 (4.8)	2 (4.7)	8 (6.2)	0 (0.0)
Second-generation antipsychotics with anticholinergic effects^a^	45 (42.9)	19 (44.2)	15 (11.6)	11 (20.0)
Second-generation antipsychotics without anticholinergic effects	63 (60.0)	26 (60.5)	16 (12.4)	6 (10.9)
Antidepressants				
Tricyclic antidepressants^a^	0 (0.0)	0 (0.0)	13 (10.1)	5 (9.1)
Tetracyclic antidepressants^a^	0 (0.0)	0 (0.0)	6 (4.7)	4 (7.3)
Selective serotonin reuptake inhibitors	4 (3.8)	2 (4.7)	31 (24.0)	12 (21.8)
Serotonin–norepinephrine reuptake inhibitors	4 (3.8)	3 (7.0)	22 (17.1)	10 (18.2)
Noradrenergic and specific serotonergic antidepressants^a^	2 (1.9)	0 (0.0)	31 (24.0)	15 (27.3)
Other	7 (6.7)	4 (9.3)	9 (7.0)	5 (9.1)
Other	10 (9.5)	3 (7.0)	14 (10.9)	4 (7.3)
Anxiolytics or sedative-hypnotics	44 (41.9)	23 (53.5)	63 (48.8)	28 (50.9)
Anxiolytics	18 (17.1)	7 (16.3)	23 (17.8)	6 (10.9)
Benzodiazepines^a^	18 (17.1)	7 (16.3)	22 (17.1)	6 (10.9)
Hypnotics	42 (40.0)	23 (53.5)	69 (53.5)	30 (54.5)
Benzodiazepines^a^	27 (25.7)	16 (37.2)	46 (35.7)	20 (36.4)
Other	2 (1.9)	2 (4.7)	5 (3.9)	4 (7.3)
Benzodiazepines^a^	2 (1.9)	2 (4.7)	5 (3.9)	4 (7.3)

Values are shown as n (%). Multiple answers are allowed.

a Medications with anticholinergic effects.

## 4 Discussion

This subgroup analysis of previously reported postmarketing surveillance data (21) indicated that elobixibat improved constipation as early as 2 weeks after treatment initiation with no new safety signal identified in patients with schizophrenia or depression and with available follow-up in real-world settings.

Although the treatment continuation rate was relatively good at approximately 80% in the 4-week treatment groups, the most common reason for treatment discontinuation or termination was lack of efficacy, which may be due to severe constipation and an insufficient elobixibat dose. In a previous single-center study of patients with constipation with concomitant mental disorders, nearly half required a 15 mg daily dose of elobixibat after 5–6 months of treatment (22). Approximately 70% to 80% of patients in the present analysis were treated with 10 mg of elobixibat instead of 15 mg. Although treatment persistence was not assessed using maximum daily dose, increasing the dose to 15 mg among patients treated with 10 mg and experiencing insufficient efficacy might have resulted in better treatment persistence. A descriptive comparison between those who completed the 52-week treatment and those who discontinued treatment suggested certain characteristics that may be associated with discontinuation of elobixibat, but their implications should be further assessed in larger studies.

Compared with previous findings in the overall population (21), patients with schizophrenia included a slightly lower proportion of women (56.2% vs. 61.1%), a lower proportion of individuals aged ≥65 years (42.9% vs. 73.7%), and a higher proportion of inpatients (49.5% vs. 9.4%), and patients with depression included a slightly higher proportion of women (69.0% vs. 61.1%), a slightly higher proportion of patients with prior use of prescription laxatives (79.8% vs. 62.8%), and a slightly higher proportion of patients with concomitant laxatives (45.7% vs. 37.5%). Despite the difference in patient characteristics, elobixibat successfully increased weekly defecation frequency and BSFS scores and improved constipation-related symptoms as early as 2 weeks after treatment initiation in the present cohort, as demonstrated in the overall population (21). As in the overall population, the proportion of patients with ADRs was < 10% in each group, and the most common ADRs were gastrointestinal disorders, particularly diarrhea. These findings support that elobixibat was safe and effective in treating constipation in patients with schizophrenia or depression.

In the present analysis, patients with schizophrenia were younger and more often treated in inpatient settings than those with depression, which may have led to a better treatment continuation rate (72.1% vs. 50.9%) with a lower treatment drop-out rate (16.7% vs. 29.6%) at 52 weeks in patients with schizophrenia than those with depression. Although weekly defecation frequency and the distribution of BSFS scores at baseline were similar between patients with schizophrenia and those with depression, the prior or concomitant use of stimulant laxatives was more common among patients with schizophrenia, indicating the severity of chronic constipation in this population. Therefore, the effectiveness and safety data in this subgroup analysis should not be compared directly between patients with schizophrenia and those with depression.

The Japanese guidelines for pharmacological therapy of schizophrenia (23, 24) state that medications with anticholinergic effects are likely to cause chronic constipation and are not recommended for use with other psychiatric medications. They also state that laxatives may be effective for constipation associated with psychiatric medications, but should be used cautiously due to possible side effects. Exercise, nutritional support, and sufficient water consumption are also recommended. The Japanese clinical practice guidelines for bowel problems state that stimulant laxatives should not be a first-choice or used regularly to avoid resistance or dependency (25). Elobixibat, which acts as both an osmotic and a stimulant agent (16) and possibly restores defecation desire (19), is considered a safe treatment option for chronic constipation among patients with schizophrenia or depression.

Aside from those previously stated in the report of the overall population (21), the present study has several limitations. First, our findings were based on a subgroup analysis of the previous postmarketing surveillance study and not on a stand-alone study targeting patients with schizophrenia or depression. Interpretation is limited by attrition, selection, and lack of a comparator. Nearly half of the patients in the 4-week treatment groups were not evaluated in the 52-week treatment groups, suggesting possible survivor bias in the 52-week findings. Second, the safety and effectiveness data of elobixibat were not stratified by the type of concomitant psychiatric medications. Third, we did not diagnose schizophrenia and depression based on study-specific criteria or evaluate their severity using scales such as the Positive and Negative Syndrome Scale and Hamilton Depression Rating Scale, resulting in potential misclassification and limiting the interpretation of effectiveness data.

## 5 Conclusion

This report is the first to analyze the effectiveness and safety of elobixibat in patients with schizophrenia or depression as part of a prospective, multicenter, postmarketing surveillance study. Our findings in the 4-week treatment groups supplement the safety and effectiveness of elobixibat demonstrated in a previous phase 3 trial (20). Given the small number of patients assessed in the 52-week treatment groups, more data are needed to support the long-term effectiveness and safety of elobixibat in this population.

## Data Availability

The raw data supporting the conclusions of this article will be made available by the authors, without undue reservation.

## Supplementary material

Supplementary Figure 1 Patient flow diagram. Safety was analyzed in the 4-week treatment group of 105 patients with schizophrenia and 129 patients with depression, as well as in the 52-week treatment group of 43 patients with schizophrenia and 55 patients with depression. Effectiveness was analyzed in the 4-week treatment group of 100 patients with schizophrenia and 120 patients with depression, as well as in a 52-week treatment group of 42 patients with schizophrenia and 51 patients with depression. CRF, case report form.

Supplementary Figure 2 Comparison between observed data and MMRM estimates. Similar trends were observed between the observed data (mean and 95% CI, shown in orange) and MMRM estimates (LS mean and 95% CI, shown in blue). CI, confidence interval; LS, least squares; MMRM, mixed model for repeated measures.

Supplementary Table 1 Baseline patient characteristics in the 52-week treatment group.

Supplementary Table 2 Defecation frequency per week in the primary and MMRM analyses.
