# Supplementary material for: Postmarketing surveillance of elobixibat for patients with chronic constipation and concomitant schizophrenia or depression in Japan
Source: Front Psychiatry. 2026 Apr 20;17:1763059. doi: 10.3389/fpsyt.2026.1763059 (PMC13136171; doi:10.3389/fpsyt.2026.1763059)
Supplement: Supplementary Figure 1 — Patient flow diagram. Safety was analyzed in the 4-week treatment group of 105 patients with schizophrenia and 129 patients with depression, as well as in the 52-week treatment group of 43 patients with schizophrenia and 55 patients with depression. Effectiveness was analyzed in the 4-week treatment group of 100 patients with schizophrenia and 120 patients with depression, as well as in a 52-week treatment group of 42 patients with schizophrenia and 51 patients with depression. CRF, case report form. [file Presentation1.pptx]

## Slide 1
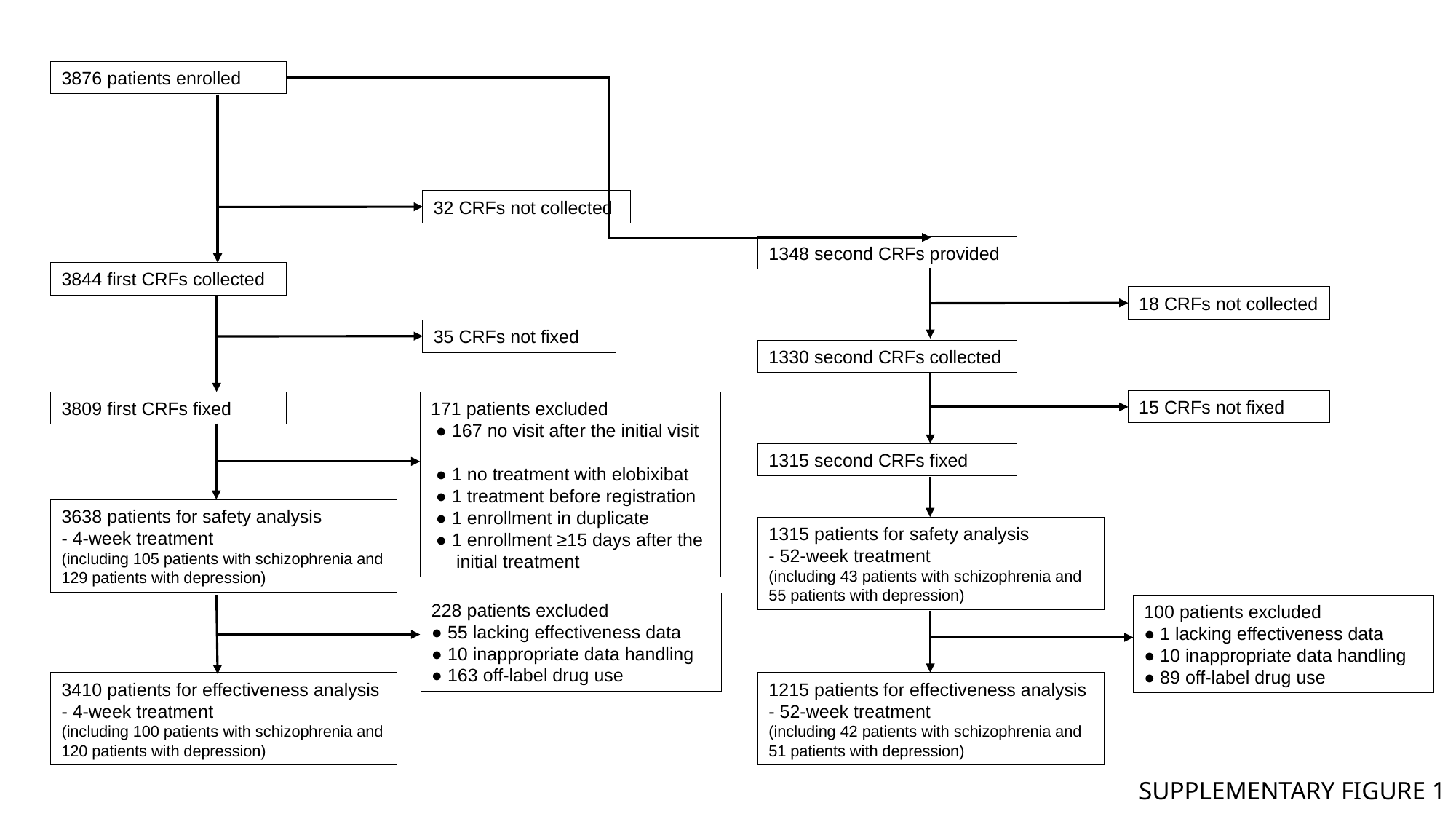

3876 patients enrolled
32 CRFs not collected
1348 second CRFs provided
3844 first CRFs collected
18 CRFs not collected
35 CRFs not fixed
1330 second CRFs collected
15 CRFs not fixed
3809 first CRFs fixed
171 patients excluded
 ● 167 no visit after the initial visit
 ● 1 no treatment with elobixibat
 ● 1 treatment before registration
 ● 1 enrollment in duplicate
 ● 1 enrollment ≥15 days after the
 initial treatment
1315 second CRFs fixed
3638 patients for safety analysis
- 4-week treatment
(including 105 patients with schizophrenia and 129 patients with depression)
1315 patients for safety analysis
- 52-week treatment
(including 43 patients with schizophrenia and 55 patients with depression)
228 patients excluded
● 55 lacking effectiveness data
● 10 inappropriate data handling
● 163 off-label drug use
100 patients excluded
● 1 lacking effectiveness data
● 10 inappropriate data handling
● 89 off-label drug use
3410 patients for effectiveness analysis
- 4-week treatment
(including 100 patients with schizophrenia and 120 patients with depression)
1215 patients for effectiveness analysis
- 52-week treatment
(including 42 patients with schizophrenia and 51 patients with depression)
SUPPLEMENTARY FIGURE 1

## Slide 2
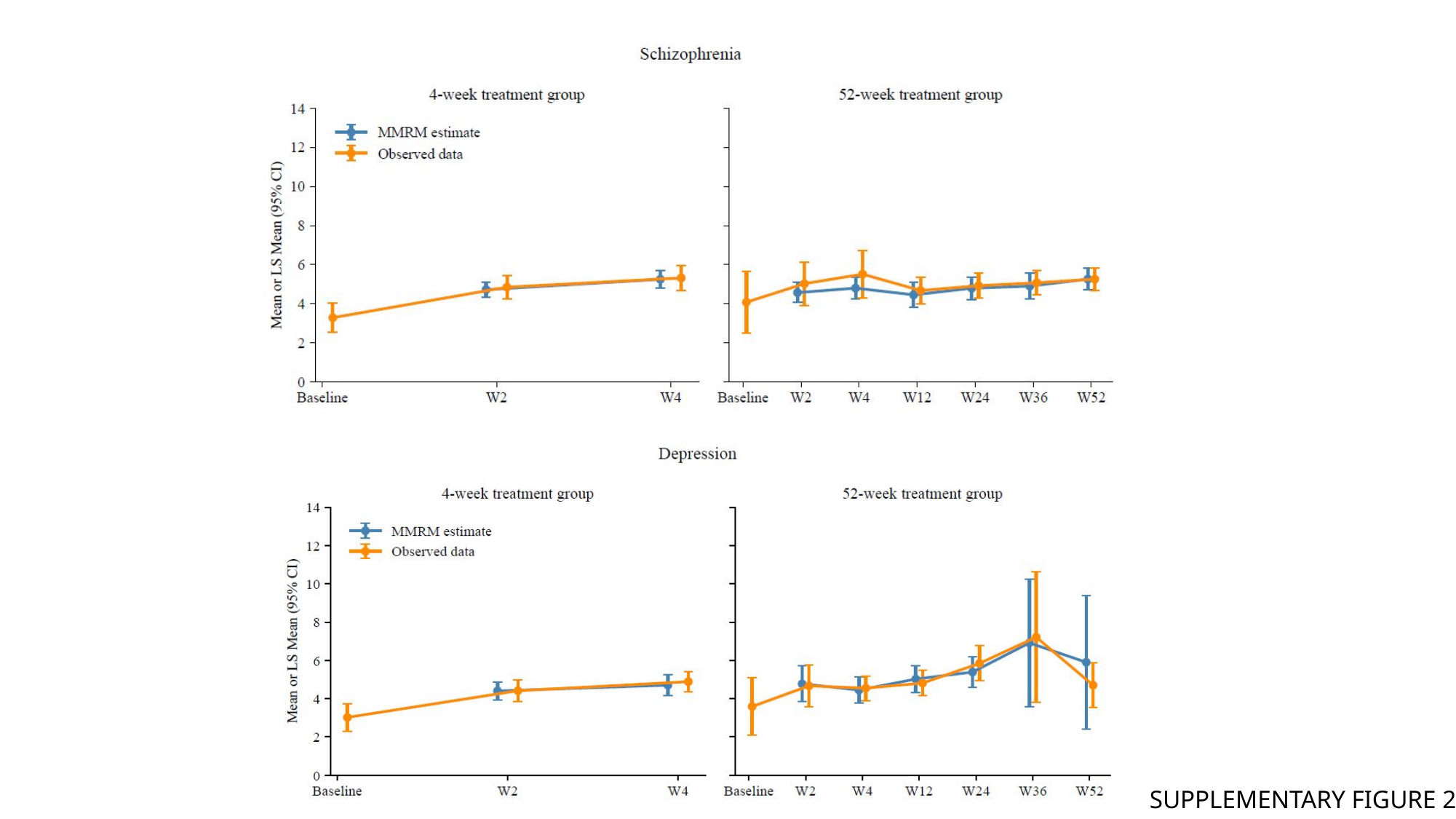

SUPPLEMENTARY FIGURE 2
