# Supplementary material for: Postmarketing surveillance of elobixibat for patients with chronic constipation and concomitant schizophrenia or depression in Japan
Source: Front Psychiatry. 2026 Apr 20;17:1763059. doi: 10.3389/fpsyt.2026.1763059 (PMC13136171; doi:10.3389/fpsyt.2026.1763059)
Supplement: Supplementary Table 1 — Baseline patient characteristics in the 52-week treatment group. [file Supplementaryfile1.docx]

Supplementary Table S1. Baseline patient characteristics in the 52-week treatment group

|  | Patients with schizophrenia  N = 43 | | Patients with depression  N = 55 | |
| --- | --- | --- | --- | --- |
|  | Completed  N = 31 | Discontinued  N = 12 | Completed  N = 28 | Discontinued  N = 27 |
| Sex |  |  |  |  |
| Men | 15 (48.4) | 4 (33.3) | 10 (35.7) | 7 (25.9) |
| Women | 16 (51.6) | 8 (66.7) | 18 (64.3) | 20 (74.1) |
| Pregnant^a^, yes | 0 (0.0) | 0 (0.0) | 0 (0.0) | 0 (0.0) |
| Age, years |  |  |  |  |
| < 65 | 15 (48.4) | 9 (75.0) | 9 (32.1) | 7 (25.9) |
| ≥ 65 | 16 (51.6) | 3 (25.0) | 19 (67.9) | 20 (74.1) |
| BMI, kg/m^2^ |  |  |  |  |
| < 18.5 | 3 (9.7) | 1 (8.3) | 4 (14.3) | 3 (11.1) |
| 18.5 to < 25 | 9 (29.0) | 5 (41.7) | 16 (57.1) | 10 (37.0) |
| ≥ 25 | 3 (9.7) | 1 (8.3) | 2 (7.1) | 3 (11.1) |
| Unknown | 16 (51.6) | 5 (41.7) | 6 (21.4) | 11 (40.7) |
| Outpatient/inpatient |  |  |  |  |
| Outpatient | 11 (35.5) | 6 (50.0) | 24 (85.7) | 24 (88.9) |
| Inpatient | 20 (64.5) | 6 (50.0) | 4 (14.3) | 3 (11.1) |
| Duration of chronic constipation, years |  |  |  |  |
| < 5 | 12 (38.7) | 3 (25.0) | 10 (35.7) | 9 (33.3) |
| ≥ 5 | 17 (54.8) | 8 (66.7) | 16 (57.1) | 15 (55.6) |
| Unknown | 2 (6.5) | 1 (8.3) | 2 (7.1) | 3 (11.1) |
| IBS-C, yes | 1 (3.2) | 3 (25.0) | 2 (7.1) | 4 (14.8) |
| Prior OTC laxative use, yes | 0 (0.0) | 1 (8.3) | 0 (0.0) | 3 (11.1) |
| Prior prescription laxative use, yes^b^ | 23 (74.2) | 10 (83.3) | 21 (75.0) | 23 (85.2) |
| Saline laxatives^c^ | 11 (47.8) | 4 (40.0) | 9 (42.9) | 12 (52.2) |
| Sugar-like laxatives^c^ | 0 (0.0) | 0 (0.0) | 0 (0.0) | 0 (0.0) |
| PEG preparations^c^ | 1 (4.3) | 0 (0.0) | 0 (0.0) | 1 (4.3) |
| Intestinal secretagogues^c^ | 3 (13.0) | 2 (20.0) | 2 (9.5) | 8 (34.8) |
| Bulk-forming laxatives^c^ | 0 (0.0) | 1 (10.0) | 0 (0.0) | 2 (8.7) |
| Stimulant laxatives^c^ | 18 (78.3) | 9 (90.0) | 10 (47.6) | 9 (39.1) |
| Other^c^ | 6 (26.1) | 1 (10.0) | 8 (38.1) | 8 (34.8) |
| Concomitant laxative use, yes^b^ | 12 (38.7) | 6 (50.0) | 12 (41.4) | 19 (73.1) |
| Saline laxatives^d^ | 8 (66.7) | 3 (50.0) | 5 (41.7) | 12 (63.2) |
| Sugar-like laxatives^d^ | 0 (0.0) | 0 (0.0) | 0 (0.0) | 0 (0.0) |
| PEG preparations^d^ | 1 (8.3) | 0 (0.0) | 1 (8.3) | 1 (5.3) |
| Intestinal secretagogues^d^ | 3 (25.0) | 2 (33.3) | 1 (8.3) | 5 (26.3) |
| Bulk-forming laxatives^d^ | 0 (0.0) | 1 (16.7) | 0 (0.0) | 1 (5.3) |
| Stimulant laxatives^d^ | 8 (66.7) | 5 (83.3) | 3 (25.0) | 7 (36.8) |
| Other^d, e^ | 3 (25.0) | 3 (50.0) | 4 (33.3) | 8 (42.1) |

BMI, body mass index; IBS-C, irritable bowel syndrome with constipation; OTC, over-the-counter; PEG, polyethylene glycol.

Values are shown as n (%).

^a^For women.

^b^Multiple responses are allowed.

^c^The proportion shown is that of patients who used any prior prescribed laxatives.

^d^The proportion shown is that of patients who used any concomitant laxatives.

^e^Including Kampo medicines and probiotics.

**Supplementary Table S2. Defecation frequency per week in the primary and MMRM analyses**

|  | Baseline | Week 2 | Week 4 | Week 12 | Week 24 | Week 36 | Week 52 |
| --- | --- | --- | --- | --- | --- | --- | --- |
| Patients with schizophrenia |  |  |  |  |  |  |  |
| 4-week treatment group | n = 84 | n = 79 | n = 81 | – | – | – | – |
| Mean (SD) | 3.3 (3.5) | 4.8 (2.7) | 5.3 (2.9) | – | – | – | – |
| 95% CI | 2.53, 4.04 | 4.25, 5.45 | 4.67, 5.97 | – | – | – | – |
| LS Mean (SE) | – | 4.7 (0.2) | 5.3 (0.2) | – | – | – | – |
| 95% CI | – | 4.33, 5.11 | 4.80, 5.70 | – | – | – | – |
| 52-week treatment group | n = 38 | n = 36 | n = 37 | n = 31 | n = 26 | n = 27 | n = 27 |
| Mean (SD) | 4.1 (4.8) | 5.0 (3.3) | 5.5 (3.6) | 4.7 (1.9) | 4.9 (1.6) | 5.1 (1.5) | 5.3 (1.5) |
| 95% CI | 2.51, 5.65 | 3.93, 6.13 | 4.30, 6.72 | 3.98, 5.38 | 4.28, 5.57 | 4.46, 5.68 | 4.67, 5.85 |
| LS Mean (SE) | – | 4.6 (0.2) | 4.8 (0.3) | 4.5 (0.3) | 4.8 (0.3) | 4.9 (0.3) | 5.3 (0.3) |
| 95% CI | – | 4.06, 5.08 | 4.23, 5.37 | 3.83, 5.08 | 4.22, 5.37 | 4.24, 5.56 | 4.71, 5.81 |
| Patients with depression |  |  |  |  |  |  |  |
| 4-week treatment group | n = 87 | n = 75 | n = 89 | – | – | – | – |
| Mean (SD) | 3.0 (3.4) | 4.4 (2.4) | 4.9 (2.4) | – | – | – | – |
| 95% CI | 2.30, 3.75 | 3.86, 4.97 | 4.37, 5.40 | – | – | – | – |
| LS Mean (SE) | – | 4.4 (0.2) | 4.7 (0.3) | – | – | – | – |
| 95% CI | – | 3.93, 4.88 | 4.16, 5.25 | – | – | – | – |
| 52-week treatment group | n = 39 | n = 30 | n = 40 | n = 32 | n = 19 | n = 19 | n = 17 |
| Mean (SD) | 3.6 (4.6) | 4.7 (2.9) | 4.6 (2.0) | 4.8 (1.8) | 5.8 (1.9) | 7.2 (7.1) | 4.7 (2.3) |
| 95% CI | 2.08, 5.10 | 3.59, 5.75 | 3.91, 5.19 | 4.16, 5.47 | 4.93, 6.75 | 3.81, 10.61 | 3.55, 5.87 |
| LS Mean (SE) | – | 4.8 (0.5) | 4.4 (0.3) | 5.0 (0.3) | 5.4 (0.4) | 6.9 (1.6) | 5.9 (1.2) |
| 95% CI | – | 3.84, 5.71 | 3.77, 5.13 | 4.33, 5.72 | 4.60, 6.18 | 3.58, 10.25 | 2.41, 9.39 |

CI, confidence interval; LS, least squares; MMRM, mixed model for repeated measures; SE, standard error.
